# Measuring the impact of cataract services in the community

**Published:** 2014

**Authors:** Sarah Polack, Hannah Kuper

**Affiliations:** Lecturer: International Centre for Evidence in Disability Group, London School of Hygiene and Tropical Medicine, London, UK. sarah.polack@Lshtm.ac.uk; Co-director: International Centre for Evidence in Disability Group, London School of Hygiene and Tropical Medicine, London, UK. hannah.kuper@Lshtm.ac.uk

**Figure:**
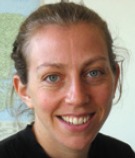
Sarah Polack

**Figure:**
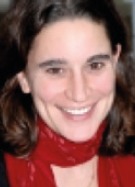
Hannah Kuper

In many low-income countries, a substantial number of people remain needlessly visually impaired or blind from cataract as a result of not accessing surgical services.^1^ In this article, we will discuss how Rapid Assessment of Avoidable Blindness (RAAB) surveys can play a role in improving cataract services, and the impact that sight-restoring cataract surgery can have on people's lives.

## How can RAAB be used to improve cataract services?

RAAB is a method for rapidly assessing visual acuity.^2^ People aged 50 years and above are randomly selected from a population. They undergo visual acuity screening and those who are found to have problems with their vision are examined by an eye care professional to determine the most likely cause. To date, more than one hundred RAAB surveys have been conducted to date across the world.

Information from RAAB surveys can be used to improve cataract services in a number of different ways.

RAAB provides estimates of the prevalence of blindness and visual impairment and its main causes. This information can be used to estimate the need for cataract surgery in the community. From this, we can estimate the number of cataract operations that need to be performed per million population per year (known as the cataract surgical rate) in order to help everyone who needs a cataract operation within a set time frame. Information is also collected on the number of people who have undergone cataract surgery; this can be used to estimate cataract surgical coverage (i.e. the proportion of patients/eyes with operable cataract who have already received surgery), which is a measure of progress.

Where no hospital data are available, RAAB survey findings about the visual acuity of people who have undergone cataract surgery can be used to give an overview of the quality of cataract services in an area or district. The causes of poor visual acuity can also be used to identify areas for improvement. For instance, if poor outcome after cataract surgery is common and is attributable to refractive error, then better optometry services may be needed. The RAAB data on quality will not be as good as hospital data in reflecting current outcomes, because RAAB will include people operated on many years ago and from a variety of different surgical services.

**Figure:**
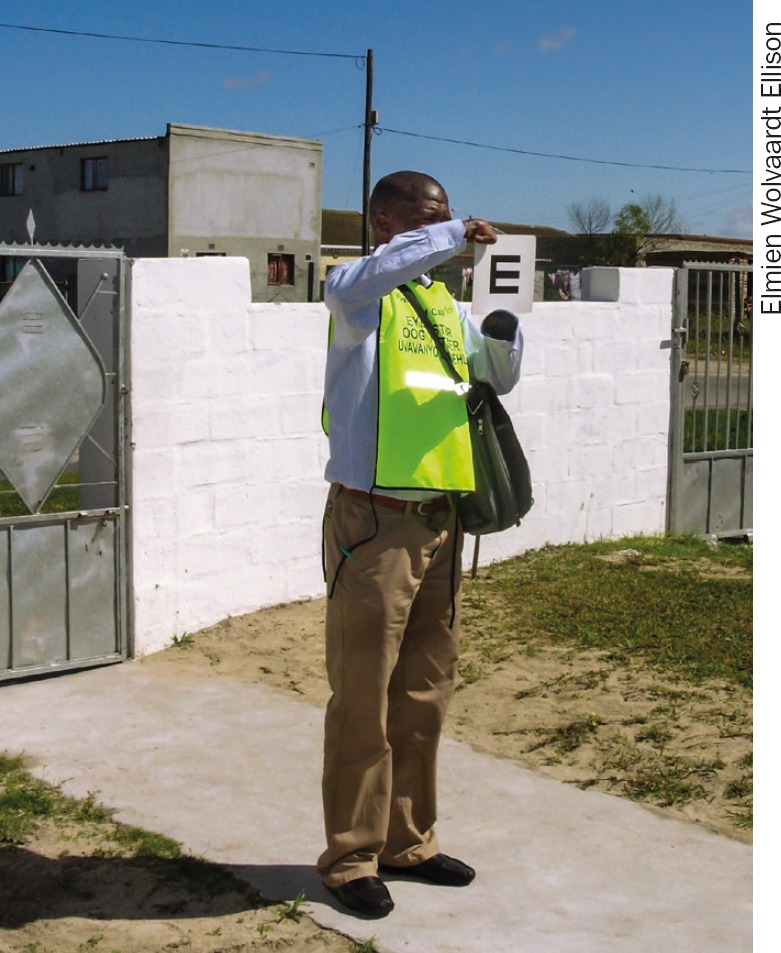
Visual acuity testing during a RAAB survey conducted in South Africa

During RAAB, people who have cataract but have not undergone surgery are asked why they have not attended. This information shows us the main barriers to overcome when providing services in an area. For instance, if cost is cited as the main barrier then the service could consider providing subsidies, whereas if lack of awareness is the main barrier then a publicity campaign may be needed.

Finally, RAAB can also be used to monitor the impact of a cataract surgery service if it is repeated after a period of time (e.g. 5–10 years) and the prevalence and causes of visual impairment at the two time points compared.

## Why is improving access to cataract services important?

It was widely believed that visual impairment from cataract could lead to poverty in the household and have a negative impact on the person, but evidence for these assumptions was lacking. We undertook the Cataract Impact Study to explore how sight-restoring cataract surgery impacts on quality of life, daily activities and poverty in Kenya, Bangladesh and the Philippines.

Using the RAAB method outlined above, as well as community-based case finding, we identified approximately six hundred people with visually impairing cataract (VA<6/24 in the better eye) across the three study countries. For each participant with cataract, we selected an age- and gender-matched peer without visual impairment (the controls). Participants and controls were interviewed about their quality of life, their daily activities and household poverty.

People with cataract were offered free or subsidised cataract surgery and one year later they were traced and interviewed again. Patients from the Philippines and Bangladesh were also followed up after six years to assess the long-term impact of surgery.

This study found that, at baseline (before surgery), when compared the controls, the people with cataract^3^:

- had poorer quality of life (both vision-related and generic)
- were less likely to engage in productive activities and were more likely to have assistance with daily activities
- were poorer in terms of household assets, self-rated wealth and monthly expenditure.

One year after cataract surgery, compared to baseline, people who had undergone cataract surgery:

- had improved vision-related and generic health-related quality of life
- were more likely to engage in productive activities and were less likely to report assistance with activities
- had significantly increased household per capita expenditure (i.e. less poverty).

Follow-up six years after surgery in the Philippines and Bangladesh found that benefits were also sustained in the long term.

Among older adults in low-income countries, this study found evidence that cataract surgery can lead to improved quality of life, greater participation and less poverty. These findings highlight the importance of providing affordable and accessible cataract surgery for all those who need it.
